# Recovery of kinematic arm function in well-performing people with subacute stroke: a longitudinal cohort study

**DOI:** 10.1186/s12984-018-0409-4

**Published:** 2018-07-18

**Authors:** Gyrd Thrane, Margit Alt Murphy, Katharina Stibrant Sunnerhagen

**Affiliations:** 1 Department of Health and Care Sciences, UiT The Arctic University of Norway, Tromsø, Norway; 2 Institute of Neuroscience and Physiology, Rehabilitation Medicine, Sahlgrenska Academy, University of Gothenburg, Gothenburg, Sweden; 3 Center for Person-Centered Care (GPCC), University of Gothenburg, Gothenburg, Sweden

**Keywords:** Stroke, Upper extremity, Kinematics, Movement analysis, Recovery, Motor impairment

## Abstract

**Background:**

Most motor function improvements in people who have experienced strokes occur within the first 3 months. However, individuals showing complete or nearly complete arm function recovery, as assessed using clinical scales, still show certain movement kinematic deficits at 3 months, post-stroke. This study evaluated the changes in upper extremity kinematics, in individuals demonstrating minor clinical motor impairments, 3–12 months post-stroke, and also examined the association between kinematics and the subjects’s self-perceived hand abilities during the chronic stage, 12 months post-stroke.

**Methods:**

Forty-two subjects recovering from strokes and having Fugl-Meyer upper extremity motor assessment scores ≥60 were included from the Stroke Arm Longitudinal Study at the University of Gothenburg (SALGOT). Kinematic analyses of a drinking task, performed 3, 6, and 12 months post-stroke, were compared with kinematic analyses performed in 35 healthy controls. The Stroke Impact Scale-Hand domain was evaluated at the 12-month follow-up.

**Results:**

There were no significant changes in kinematic performance between 3 and 12 months, post-stroke. The patients recovering from stroke showed lower peak elbow extension velocities, and increased shoulder abduction and trunk displacement during drinking than did healthy controls, at all time points. At 12 months, post-stroke, better self-perceived arm functions correlated with improved trunk displacements, movement times, movement units, and time to peak velocity percentages.

**Conclusion:**

Kinematic movement deficits, observed at 3 months post-stroke, remained unchanged at 12 months. Movement kinematics were associated with the patient’s self-perceived ability to use their more affected hand.

**Trial registration:**

ClinicalTrials: NCT01115348.

## Background

Post-stroke upper extremity motor deficits are common, and initially range from total paralysis to near full upper extremity function [1, 2]. Many patients improve substantially during the first 4 post-stroke weeks, with the majority of improvements occurring within the first 3 months [3–5]. Upper extremity motor function improvements beyond the sub-acute stage might be induced by rehabilitative therapies [6–8], but the improvement observed using clinical scales is small during the sub-acute stage, compared with that occurring during the acute stage [6]. This finding may partly be attributed to the ceiling effect associated with clinical scales, such as the Fugl-Meyer Assessment upper extremity motor part (FMA-UE) [9]. More accurate motor control measurements might guide more precise treatment and more accurate monitoring of treatment results.

A kinematic analysis, using multiple optoelectronic high-speed cameras, provides objective and detailed measurement of movement quality and performance during reaching tasks [10–13]. The kinematic characteristics measured post stroke include movement time, smoothness, and velocity, as well as joint angles and movement strategies.

Post stroke motor and sensory impairments might lead to increased numbers of corrections and speed alterations during upper extremity movements, impairing movement smoothness [14] and resulting in longer completion times [15]. Adequate velocity control is essential for the quality of post-stroke arm movements, and requires intact feed-forward and feedback systems [10]. Individuals recovering from strokes take longer to complete reaching tasks. Additionally, the peak velocity appears later and they spend relatively more time in the deceleration phase of reaching than do healthy people [12, 16, 17]. These individuals also extend their elbows less during reach-to-grasp movements, and the elbow joint peak angular velocity discriminates well between people suffering from mild to moderate strokes and healthy controls [12]. Post-stroke motor impairments might also lead to compensatory shoulder or trunk movements during upper extremity tasks [12, 18, 19]. The increased trunk movement during reaching can be part of a strategy to improve the control, speed, and smoothness of arm and hand movements as well as to compensate for reduced elbow extension [19–21]. Further, compensatory shoulder movement patterns might be related to abnormal recruitment of the shoulder abductor muscles [13, 19, 22, 23].

Three months post-stroke, individuals with submaximal upper extremity FMA-UE scores (60–65 points) have lower tangential and angular peak velocities and use more trunk displacement than do healthy controls. Furthermore, individuals with full FMA-UE scores, 3 months post stroke, do not demonstrate the same elbow angular velocities as do healthy controls. They also use more trunk movements during reaching and demonstrate greater arm abduction during drinking [24]. However, whether the remaining movement deficits resolve over time remains unclear. Previous research have shown improvement that the Fugl Meyer upper extremity score might improve even after 3 months in subgroups of patients [25]. Because movement quality might improve due to targeted exercise during the chronic stage of stroke recovery [26, 27], we hypothesized that kinematic performance might improve, in well-performing individuals, later than 3 months post stroke.

Few studies have examined how kinematic measures relate to self-perceived arm and hand functioning during the performance of daily activities [28]. Therefore, the extent to which kinematic performance deficits are related to patient-perceived functional problems is also uncertain. Thus, this study examined kinematic movement deficits, between 3 and 12 months post-stroke, in individuals with complete or near-complete recovery, as assessed using the FMA-UE, and examined the degree to which the kinematic performance at 12 months was associated with a patient’s self-perceived ability to use their more affected arm.

## Methods

### Study design

The Stroke Arm Longitudinal Study at the University of Gothenburg (SALGOT) consisted of a cohort of patients recovering from first-time strokes who were admitted to the stroke unit at Sahlgrenska University Hospital during an 18-month period in 2009–2010. The present study included only those study patients who had experienced unilateral ischemic or hemorrhagic strokes, were ≥ 18-years-old, demonstrated upper extremity impairment at 1 or 2 days post-stroke (Modified Motor Assessment Scale score < 15) [29], and had FMA-UE scores ≥60 at 3 months post-stroke. The included patients were evaluated at 3, 6, and 12 months post-stroke. In addition, 35 healthy controls who considered themselves in good health and did not have any medical conditions affecting their arm functions were also recruited for kinematic testing.

### Kinematic analysis

A motion capture system (ProReflex MCU240 Hz, Qualisys, Gothenburg, Sweden), with five optoelectronic cameras, was used to obtain movement data during a standardized drinking task [12]. The task included five phases: reaching and grasping the glass, lifting the glass from the table to the mouth, taking one sip of water, placing the glass behind a line marked on the table, and returning the glass to its initial position. Participants were instructed to sit against the back of a chair during the whole task, but they were not restrained in that position. In the initial position, the participants sat close enough to reach the drinking glass using their less-affected arm, without trunk displacement. The pronated hand rested on the table, with the wrist line close to the edge of the table. The chair height was adjusted so that, in the sitting position, the participant demonstrated 90° of flexion in the hips and knees; the table height was adjusted so that the forearm was placed in the horizontal plane with the elbow at 90° of flexion. Retroreflective markers were placed on the third metacarpophalangeal joint of the hand, styloid process of the ulna on the wrist, lateral epicondyle of the elbow, middle part of the acromion on the right and left shoulders, upper part of the sternum, forehead, and the upper and lower edges of the glass. The task was performed five times at a comfortable speed, with the mean time of the 3 middle trials used for the analysis [30]. Data were captured using the more affected arm of the patients recovering from stroke and using the non-dominant hand of the healthy controls.

Endpoint variables were calculated from the hand marker and included the total movement time (s), number of movement units (n), peak velocity during reaching (cm/s), and time to peak velocity percentages (%). The total movement time included the time required to complete the whole drinking task and was measured from the point where the hand marker surpassed 2% of the peak velocity of the reaching phase through to the point where it returned back to 2% of the peak velocity of the returning phase. The number of movement units reflected the number of velocity peaks during the task, excluding the drinking phase [31]. A difference between the local minimum and the next maximum velocity value that exceeded the 20 mm/s amplitude limit signified a velocity peak. The time between two subsequent peaks had to be at least 150 ms to be defined as a movement unit [12]. The minimum number of movement units was 4, one for each movement phase. The peak hand velocity and the time to the peak velocity percentage during reaching, indicating the relative acceleration time and initial movement effort, were calculated.

Elbow angles were calculated between the vectors joining markers at the wrist and elbow, and at the elbow and shoulder. The joint angle at maximal extension and the peak angular velocity of the elbow joint during reaching were computed. Trunk displacement was defined as maximal forward displacement (mm) of the sternal marker from the initial position during the entire task. Shoulder abduction in the frontal plane, while drinking, was defined as the maximal angle between the vector joining the shoulder and elbow markers, and the vertical vector from the shoulder marker [12].

### Clinical scales

Motor impairment of the more affected arms of patients recovering from strokes was assessed using the FMA-UE. The FMA-UE includes 33 items that assess movement, coordination, and reflex actions of the shoulder, elbow, forearm, wrist, and hand. Each item is scored on a 3-point ordinal scale (0, cannot perform; 1, performs partially; and 2, performs fully); the test has a total possible score range of 0–66 [32]. The sensations for light touch and position were assessed using the FMA domain for sensation (FMA-Sensation), which has a total score range of 0–12; a score of 12 indicates normal sensation [32]. Covariates included age and body height. For the stroke group, we gathered information regarding the stroke type (hemorrhagic or ischemic) and the time elapsed since the stroke. The stroke severity was assessed using the National Institutes of Health Stroke Scale (NIHSS) score at the time of hospital admission. NIHSS scores range from 0 to 42, with a score < 6 indicating a mild stroke and a score > 20 indicating a severe stroke.

The Stroke Impact Scale (SIS) is a 64-item, self-reported questionnaire divided into eight domains [33]. The five hand function domain items were used to quantify the patient’s self-perceived ability to use the more affected arm to carry heavy objects, turn a door knob, open a can or jar, tie a shoelace, or pick up a coin. Each item was rated on a 5-point Likert-scale, with 5 indicating no difficulties and 1 indicating that the activity could not be performed. Data from the 12-month assessment were used in the analysis, with the results being presented as percentages of the maximum score. The reliability of the SIS hand domain (SIS-Hand) is reported to be adequate, valid, and sensitive to changes in patients recovering from strokes [33].

### Statistical analyses

The statistical analyses were performed using R language for statistical computing, version 3.4.2 [34]. The distribution of the included variables was evaluated by visually inspecting histograms, qq-plots, and boxplots. Normally distributed variables are described using means and standard deviations (SD); non-normally distributed variables are described using medians and quartiles.

Longitudinal changes were assessed using linear mixed models in the Lme4 package [35]. Fixed effects included time and the cofactors of age, body height, and FMA-UE score. The participants were grouped according to their FMA-UE results into either the submaximal (FMA-UE, 60–65 points) or maximal (FMA-UE, 66 points) subgroup. Random effects included the inter-subject variation of intercept and inter-subject variation in change across the three time points. Eight different analyses were run for the eight dependent variables. A step-up strategy was used to evaluate which factors contributed significantly to the model [36]. Model selection started with an intercept-only model that included both random and fixed intercepts. Because development over time was the primary interest, a time model was constructed first. The random effect of time was tested first, followed by the fixed effect of time. An adjusted model was then built by adding the fixed effect of the FMA group, followed by age, body height, side of stroke lesion and the concordance between the dominant hand and the affected side. At each step of model building, the log-likelihood ratio test was used to determine the significance of each inserted variable. The restricted maximum likelihood criteria were used for testing random effects, whereas the maximum likelihood criteria were used to test the significance of the fixed factors [36]. Variables that contributed significantly (*p* < 0.05) to the model at each step were included [36]. The time and adjusted models were then run without outliers (defined as more than 3 SDs away from the mean) to assess their potential effect on the results. Because most of the kinematic variables had at least one outlier, the Wilcoxon rank-sum test was used to determine the differences between the kinematic variables in both the patients and healthy controls. The location difference (LD), which reflects the median difference between groups, was used to describe the effect size [37]. The *p*-values from the 24 comparisons (8 dependent variables × 3 time points) were adjusted for the false discovery rate using the Benjamini-Hochberg method [38]. The association between the SIS-Hand and kinematic variables, at 12 months post-stroke, was analyzed using Spearman’s correlation.

The power of the linear mixed models was calculated using the Simr package [39]. Because the data were already available, we used the variance components from the actual data for the power calculations. The lowest detectable effect size was set to 0.75 SDs during the 9-month follow-up period. The power of detecting a true change, over time, of more than 0.75 SDs was > 95% in all models.

## Results

**Fig. 1:**
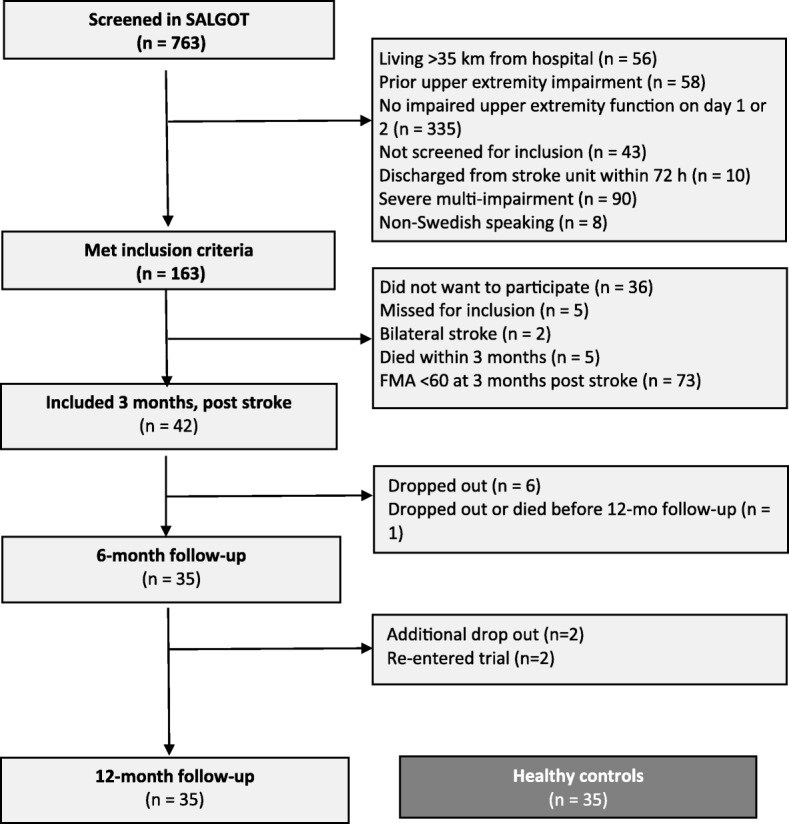
Study inclusion process and the samples taken for analysis

Forty-two subjects met the inclusion criteria for this study. Figure 1 shows the flow chart of the inclusion process. The included patients were younger (*p* = 0.003) and suffered less severe strokes, according to their admission NIHSS scores (*p* < 0.001), than were the SALGOT patients not meeting the inclusion criteria. Mild and moderate strokes were predominant, although the admission NIHSS scores ranged between 0 and 24. Table 1 shows the characteristics of the included participants.

**Table 1:** Demographic and clinical characteristics of the study population

	Stroke Group	Healthy Group
Admission	(*n* = 42)	(*n* = 35)
Age, mean (SD)	64.9 (12.2)	60.1 (12.7)
Female, n (%)	16 (38%)	15 (42%)
Ischemic stroke, n (%)	32 (87%)	
Stroke location, n (%)		
* Left hemisphere	19 (45%)	
* Right hemisphere	23 (55%)	
Dominant arm affected	21 (50%)	
NIHSS, median (QQ)	4 (3–6)	
3 days post-stroke		
FMA-UE, median (QQ)	56 (43–62)	
FMA-Sensation, *n* > 12	10	
Physical or occupational therapy	42 (100%)	
> 3 times/week 100%	42 (100%)	
3 months post-stroke		
FMA-UE, median (QQ)	65 (64–66)	
FMA-Sensation, n > 12	2	
Physical or occupational therapy	22 (52%)	
< 2 times/week	10 (24%)	
2–3 times/week	11 (26%)	
> 3 times/week	1 (2%)	
6 months post-stroke (*n* = 35)		
FMA-UE, median (QQ)	66 (63–66)	
FMA-Sensation, n > 12	1	
Physical or occupational therapy	11 (31%)	
< 2 times/week	5 (14%)	
2–3 times/week	5 (14%)	
> 3 times/week	1 (3%)	
12 months post-stroke (n = 35)		
FMA-UE, median (QQ)	66 (62.5–66)	
FMA-Sensation, n > 12	2	
SIS-Arm ability, median (QQ)	95 (78–100)	
Physical or occupational therapy	6 (17%)	
< 2 times/week	4 (11%)	
2–3 times/week	2 (6%)	
> 3 times/week	0	

SD, Standard deviation; QQ, 1st Quartile–3rd Quartile; NIHSS, National Institutes of Health Stroke Scale; FMA–UE, Fugl-Meyer Upper Extremity Motor score; FMA-Sensation, the upper extremity part of the Fugl-Meyer–Sensation score; SIS, Stroke Impact Scale

**Fig. 2:**
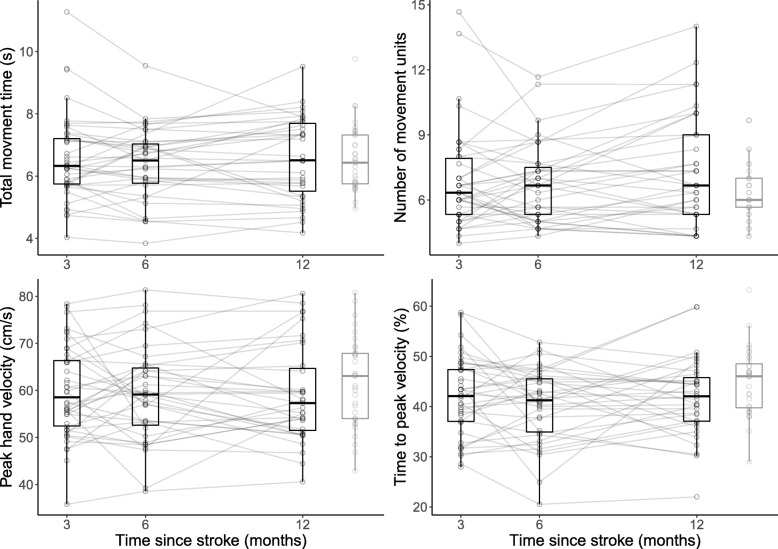
Change of endpoint variables between 3 and 12 months in post-stroke patients compared with healthy controls

**Fig. 3:**
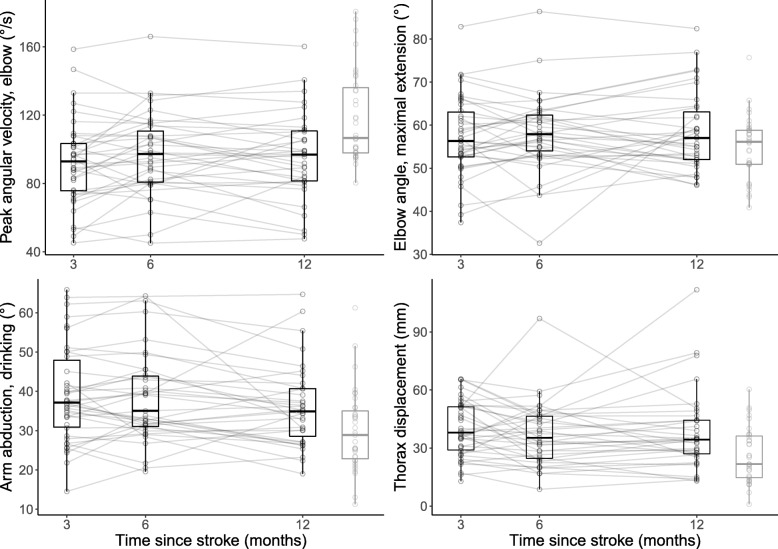
Changes of the elbow, shoulder, and trunk variables between 3 and 12 months in post-stroke patients compared to healthy controls

Figures 2 and 3 show the longitudinal changes in the kinematic variables; the descriptive characteristics of the kinematic variables are shown in Table 2. The fixed effects of time on kinematic variables are shown in Table 3; the effect sizes were close to 0 for the 8 variables. Only the number of movement units showed a small, statistically significant increase over time (*p* = 0.042) in the time- and adjusted models. The random effects analysis showed that the variance of movement time (variance [V], 0.01; covariance [COV], − 0.05; *p* = 0.006) and arm abduction (V, 0.76; COV, − 5.99; *p* = 0.005) decreased over time. The trunk displacement variance showed a small increase over time (V, 0.58; COV, 7.69; *p* = 0.041). Table 3 also shows the effects of time, adjusted for covariates that were significant in the multivariate analysis. The multivariate analysis showed that participants with maximal FMA-UE scores demonstrated shorter movement times (− 1.40 s; standard error [SE], 0.31 s), fewer movement units (− 2.08; SE, 0.55), higher peak velocities (10.91 cm/s; SE, 2.20 cm/s), and higher peak elbow angular velocities (17.66°/s; SE, 5.89°/s) than those with submaximal scores (60–65). Increased body height resulted in lower elbow peak angular velocities (− 1.13°/s; SE, 0.32°/s) and larger elbow angles (less extension) at the point of maximal extension. Subjects with a left side stroke lesion showed less arm abduction during drinking (− 7.3°, SE 3.2°), compared to right side stroke lesion. There were only minor influences on the effect of time.

**Table 2:** Descriptive statistics for the kinematic analyses

	Stroke Group			Healthy controls (mean (SD))
	3 months (mean (SD))	6 months (mean (SD))	12 months (mean (SD))	
N	42	35	35	35
Movement time, s	6.58 (1.38)	6.37 (1.18)	6.59 (1.32)	6.55 (1.03)
Movement units, n	6.86 (2.26)	6.76 (1.84)	7.33 (2.54)	6.43 (1.43)
Peak hand velocity, cm/s	59.74 (9.67)	59.26 (10.28)	59.15 (10.26)	61.93 (9.32)
Time to peak velocity, %	42 (8)	40 (7)	42 (8)	45 (7)
Elbow peak angular velocity, °/s	92.24 (24.26)	97.14 (24.37)	95.87 (25.12)	118.58 (26.43)
Elbow angle, °	57.16 (9.19)	58.23 (8.92)	58.43 (9.04)	54.78 (7.21)
Arm abduction, °	39.18 (12.31)	37.97 (11.15)	36.05 (10.44)	29.97 (10.35)
Trunk displacement, mm	39.81 (14.19)	36.96 (16.53)	38.23 (20.40)	26.19 (1.03)

*SD* Standard deviation. Velocity variables are measured in the reaching phase. The elbow angle is the minimum elbow angle in reaching indicating maximum extension of the elbow joint. Arm abduction during is measured during the drinking phase

**Table 3:** Fixed effect of time on kinematic performance changes between 3 and 12 months, post-stroke

Kinematic variable	Time β (SE)	Time Adjusted model β (SE)
Movement time (s)	0 (0.02)^b^	0 (0.02)^b,c^
Movement units (n)	0.06 (0.03)^a^	0.06 (0.03)^b, c, a^
Peak hand velocity (cm/s)	0 (0.15)	0 (0.15)^c^
Time to peak velocity (%)	0 (0)	
Elbow peak angular velocity (°/s)	0.31 (0.27)	0.30 (0.28)^c,d^
Elbow angle, maximum extension (°)	0.11 (0.12)	0.13 (0.12)^d^
Arm abduction (°)	−0.32 (0.17)^b^	− 0.32 (0.17)^b,e^
Trunk displacement (mm)	−0.09 (0.30)^b^	

^a^*p*-value > 0.05

^b^Adjusted for the random effect of time

^c^Adjusted for the maximal/submaximal Fugl-Meyer Upper Extremity Motor score

^d^Adjusted for body height

^e^Adjusted for stroke location (left/right). Velocity variables are measured in the reaching phase. The elbow angle is the minimum elbow angle in reaching indicating maximum extension of the elbow joint. Arm abduction during is measured during the drinking phase

Figures 2 and 3 also displays the differences between the kinematic variables in the recovering patients and the healthy controls. There were no cross-sectional differences between these groups of individuals with respect to the total movement time, number of movement units, or peak hand velocity at any of the measured time points. There tended to be a shorter relative time to the peak velocity among recovering patients than among healthy controls, but this was only statistically significant at 6 months, post-stroke (LD, − 3.137%; *p* = 0.039). The elbow peak angular velocity was significantly lower in the patients at 3 (LD, − 23.9^o^/s; *p* = 0.001), 6 (LD, − 18.2^o^/s; *p* = 0.006), and 12 months post-stroke (LD, − 18.9^o^/s; *p* = 0.026). The recovering patients used more arm abduction while drinking at 3 (LD, 8.7^o^; *p* = 0.003), 6 (LD, 7.6^o^; *p* = 0.013), and 12 months (LD, 5.7^o^; *p* = 0.026) post-stroke than did the healthy controls. Similarly, they also demonstrated more trunk displacement at 3 (LD, 14 mm; p = 0.001), 6 (LD, 10 mm; *p* = 0.016), and 12 months post-stroke (LD, 10 mm; *p* = 0.019) than did the healthy controls. At 12 months, the SIS-Hand score distribution for those recovering from strokes was skewed towards higher values, with the majority (20/35) reporting no difficulties in any hand item or only a little difficulty using the hand in a single item; their total scores were 95–100. In the whole stroke group, a higher SIS-Hand score was associated with shorter movement times (*ρ* = − 0.376, *p* < 0.001), fewer movement units, (*ρ* = − 0.295, *p* = 0.002), higher time to peak velocity percentages (*ρ* = 0.38, *p* < 0.001), and less trunk displacement (*ρ* = − 0.205, *p* = 0.035).

## Discussion

This study evaluated the development of upper extremity kinematic performance between 3, 6, and 12 months post-stroke in patients exhibiting only minor clinical upper extremity impairment. Three months post stroke, the total movement time, the number of movement units, the peak velocity, the time to peak velocity, and the elbow angle at maximal extension had reached a level similar to healthy controls. A small increase in the number of movement units was observed, indicating a possible but clinically insignificant decline in movement quality between 3 and 12 months. The peak angular velocity of the elbow, arm abduction during drinking and forward trunk displacement was significantly different from healthy controls at 3 months post stroke. From 3 to 12 months post stroke there was no development and the recovering patients still showed lower elbow peak angular velocity and more forward trunk movement during reaching than did the healthy controls; they also used more arm abduction during drinking. Twelve months post-stroke, improved movement times, movement units, and relative time to peak velocity values during reaching were associated with improved abilities to use the more affected arm.

To our knowledge, this is the first study to show upper extremity kinematic deficits that do not resolve within 12 months, post-stroke, in high-functioning individuals recovering from strokes. Previous cohort studies of arm kinematics have evaluated movement time and different measures smoothness up to 3 months after stroke [40–42]. However, the longitudinal course of trunk displacement, peak angular velocity of the elbow and arm abduction during drinking has not been evaluated. The deficits seen in trunk, shoulder, and elbow movement control may be part of a synergetic pattern commonly observed in more severely affected stroke survivors. Individuals with upper extremity impairments use fewer joint combinations during pointing movements than do healthy controls [43], and the contribution of elbow extension during reaching is often reduced [18]. A pathological coupling between the shoulder and elbow that reduces the degrees of freedom has been proposed [44–46], and there is evidence for this coupling in kinematic [13, 44] and electromyographic studies [23]. Another typical way of compensating for impaired elbow or shoulder function is to use the trunk to improve speed, available distance, movement quality, and precision [21, 47–49]. Whereas the upper extremity is predominantly activated through contralateral corticospinal pathways, the trunk muscles are activated bilaterally [50, 51]. Therefore, trunk movements might be more readily available and used during reaching to compensate for upper extremity impairments, such as limited elbow extension velocity. A novel finding of the present study is that high-functioning individuals recovering from strokes also use alternative strategies to compensate for minor movement deficits, and they rely on these strategies for at least 12 months, post-stroke.

In this study, patients recovering from strokes reached levels similar to those of the healthy controls for the endpoint kinematic measures of movement time, smoothness, and time to peak velocity at 3 months, and no further improvements were observed. This agrees with previous research that showed improvements in movement time and smoothness up to 6 or 8 weeks after stroke, and little or no improvement between 8 and 26 weeks [40, 41]. Although the recovering patients did not differ significantly from the controls with respect to movement times and numbers of movement units, these variables correlated with self-perceived arm ability (SIS-Hand) at 12 months, post-stroke. However, this association was weak, possibly due to the large variability in movement times and units for individuals in the stroke group, especially among those who scored ≥95% on the SIS-Hand evaluation. The correlation between the increased time to peak velocity percentages and self-perceived hand ability should be interpreted in light of the tendency towards lower percentages among individuals in the stroke group than among the healthy controls. Among the kinematic variables that were significantly different between the patients and controls, increased trunk displacement associated with the stroke group showed a weak but significant correlation with difficulties using the more affected arm. The correlations between kinematic variables and self-perceived hand abilities, shown in this study, are similar in size to correlations previously shown using the ALBILHAND measure [28]. Even small to moderate correlations indicate that minor kinematic performance deficits might relate to the perceptions that these individuals have of their ability to use their affected hands during functional tasks.

Several reasons might explain why some of the movement deficits do not resolve within the first year, post-stroke. The post-stroke period between 3 and 12 months is outside the critical period for neurobiological improvement [6]. This and the lower level of rehabilitation provided by health care systems beyond the first 3 post-stroke months, especially for well-performing patients, might explain the plateau observed for some kinematic measures. On the other hand, since well-functioning patients are able to use their affected upper extremities during daily activities, movement performance might be expected to reach a level similar to that of healthy controls during the first year, post-stroke. In the present study, this was true for some endpoint kinematics, such as movement time and smoothness, but not for the elbow angular velocity and compensatory arm and trunk movements. An explanation for this finding might be that slowness and clumsiness are more easily perceived by the patient. Thus, the demands of daily life may constantly force the recovering patient to improve their functioning, whereas minor modifications in arm and trunk movements may become habitual and difficult to address without professional guidance. Because high-functioning stroke survivors succeed in their most important activities by using compensatory movements, they may not be motivated to improve minor deficits in their movement patterns [52, 53].

This study has some limitations that should be acknowledged. The results from the current study are most relevant for people suffering from mild stroke impairments. Further, the results of our kinematic analyses are specific to the drinking task [54]. However, the use of a common purposeful task, such as drinking, improves the ecological validity of the results. The functional task used in this study included drinking, making it inappropriate to repeat numerous trials. However, the use of the average from more than 3 attempts could possibly have improved the stability of the kinematic analysis. Since patients in the stroke group performed the kinematic testing several times, unlike the controls, a learning effect cannot be ruled out; however, it is probably negligible due to the number of repetitions needed to improve upper extremity movement kinematics [13]. The relatively high dropout rate at 12 months (16%) might impact the external validity of the results. In addition, the outliers might have impacted the results, although several measures were taken to control for this impact. A ceiling effect was present in the SIS-Hand score, indicating that most of the patients in the stroke group had reached adequate hand ability or that the SIS-Hand evaluation was insufficiently sensitive to changes at 12 months, post-stroke.

## Conclusions

This study evaluated changes in upper extremity movement kinematics in high-functioning individuals between 3 and 12 months, post-stroke, and examined the association of these changes with the patients’ self-perceived ability to use their most affected hands during the chronic stage of stroke recovery. Our results show that little or no change in kinematic performance occurred between 3 and 12 months, post-stroke. The endpoint measures reached levels similar to those of the healthy controls within the first 3 months after the stroke. Further, the impairment of the elbow extension velocity and the altered movement patterns of the arm and trunk did not resolve during the 3–12-month post-stroke period. Compensatory movement patterns may help patients succeed in performing basic activities, but may also limit the possibilities for retraining the affected arm during daily activities and lead to more permanent movement changes [46]. The correlation between the patient’s self-perceived ability to use their more affected hand and the patient’s observed kinematic performance indicated that kinematic performance deficits might be clinically important. Previous research has demonstrated the possibility of retraining kinematic performance in people with mild stroke impairment [26]. Thus, depending on the complexity of a patient’s goals, we recommend the evaluation of kinematic performance in these individuals to help develop individually targeted interventions.

## Abbreviations

COV: covariance
FMA: Fugl-Meyer Assessment
FMA-UE: Fugl-Meyer Assessment – upper extremity
LD: location difference
NIHSS: National Institute of Health Stroke Scale
SD: standard deviation
SE: standard error.
SIS: Stroke Impact Scale
SOLGAT: Stroke Arm Longitudinal study at the University of Gothenburg
V: variance
